# Effect of Solvent Variations in the Alcothermal Synthesis of Template-Free Mesoporous Titania for Dye-Sensitized Solar Cells Applications

**DOI:** 10.1371/journal.pone.0164670

**Published:** 2016-10-14

**Authors:** Maciej Zalas, Agata Wawrzyńczak, Paulina Półrolniczak, Jan Sobuś, Grzegorz Schroeder, Stefan Jurga, Elena Selli

**Affiliations:** 1 Faculty of Chemistry, Adam Mickiewicz University in Poznań, Umultowska 89 B, 61–614, Poznań, Poland; 2 Department of Chemistry, University of Milan, Via Golgi 19, Milano, 20133, Italy; 3 Institute of Non-Ferrous Metals Division in Poznań Central Laboratory of Batteries and Cells, Forteczna 12, 61–362, Poznań, Poland; 4 NanoBioMedical Centre, Adam Mickiewicz University in Poznań, Umultowska 85, 61–614, Poznań, Poland; 5 Quantum Electronics Laboratory, Faculty of Physics, Adam Mickiewicz University in Poznań, Umultowska 85, 61–614, Poznan, Poland; Institute of Materials Science, GERMANY

## Abstract

A series of 14 mesoporous titania materials has been synthesized using the simple alcothermal template-free method and various alcohols, such as methanol, propanols and butanols, as solvents. All materials were characterized by both wide and small angle XRD, which exhibited the anatase phase with short-range ordered mesoporous structure that is still forming during post synthetic temperature treatment in most of the investigated materials. Nitrogen adsorption–desorption isotherms confirmed the mesoporous structure with surface area ranging from 241 to 383 m^2^g^- 1^ and pore volumes from 0.162 to 0.473 m^3^g^-1^, UV-Vis diffuse reflectance showed the redshift of the absorption edge and the bandgap decrease after post synthetic calcination of the materials presented. The TEM, FT-IR, DTA and TG measurements have been made to well characterize the materials synthesized. The mesoporous samples obtained were applied as anode materials for dye-sensitized solar cells and showed good activity in photon-to-current conversion process with efficiency values ranging from 0.54% to 4.6% and fill factors in the 52% to 67% range. The photovoltaic performances were not as high as those obtained for the materials synthesized by us earlier employing ethanol as a solvent. The differences in the electron lifetime, calculated from electrochemical impedance spectroscopy results and varying between 4.3 to 17.5 ms, were found as a main factor determining the efficiency of the investigated photovoltaic cells.

## 1. Introduction

Dye-sensitized solar cells (DSSC) are well performing cheap devices that produce green energy by direct conversion of solar light into electricity [1]. A typical DSSC consists of two sheets of transparent conductive oxide (TCO) coated glass. One of them, the so-called working electrode, is covered with the mesoporous electrode, mostly made of mesoporous nanocrystalline TiO_2_, sensitized with a synthetic or natural dye [2]. The second TCO glass sheet, the so-called counter electrode, is covered by a thin layer of platinum. These two electrodes are arranged in a sandwich cell and the space between them is filled with a liquid electrolyte containing the I^-^/I_3_^-^ redox pair in organic solvent(s).

DSSC had been investigated long before O’Regan and Grätzel presented their highly-effective device. Initially TiO_2_ with smooth surface was used as anode material and, as a consequence, low dye adsorption took place, which led to efficiencies below 1%. The critical invention was the application of nanocrystalline mesoporous TiO_2_ as an electrode material, which resulted in an over 1000 times increase in the dye load and an almost 10 times better photoconversion performance in the O’Regan and Grätzel cell [1, 3]. After Zukalová et al. reported that the DSSC electrode material consisting of an organized mesoporous titania film showed a solar energy conversion efficiency enhanced by about 50% if compared with non-ordered ones [4], the interest in the synthesis and application in DSSC of well-ordered mesoporous titania increased significantly.

The typical synthesis of mesoporous titania with both amorphous and crystalline walls framework involves the presence of various types of templates to control the shape and structure of the pores [5], though a few reports appeared in the literature about the successful synthesis of mesoporous titania without using any template material. Qi et al. obtained well-crystallized anatase with disordered mesoporous structure using only the titanium precursor in ethanolic solution and distilled water [6]. Mesoporous titania with crystalline framework obtained via controlled hydrolysis at room temperature of titanium(IV) n-butoxide in the presence of a small amount of nitric acid catalyst was reported by Liu et. al [7]. The as-prepared material exhibited a nanoporous and amorphous structure and its subsequent temperature treatment led to the formation of well-crystallized anatase with disordered mesoporous structure. The simultaneous formation of macro- and mesopores in sponge-like structures was further reported [8–10]. In general, such structures were obtained via dropwise addition of titanium precursors to deionized water or water solutions of a weak inorganic acids, followed by calcination of the obtained precipitate after various ageing times. Raveendran et. al. first reported a short range ordered mesoporous anatase, with spherical morphology, prepared via a template-free method [11]. The ordered structure was obtained via hydrolysis of titanium(IV) n-butoxide in ethyl acetate after addition of a small amount of water. More recently the template-free methodology of mesoporous titania synthesis is focusing on thin films (both, bare [12, 13] and doped [14, 15]) and three-dimensional structures [16–20]. Numerous template-free mesoporous titania materials have been applied as active photocatalysts [9, 10, 21–23] or electrode materials in DSSCs [24, 25]. The template free synthesis of the mesoporous materials seems to be worth of further investigation, when taking into account that it is environmentally friendly, cheaper and often needs milder synthesis conditions.

In this work we present the development of the alcothermal template-free synthesis of mesoporous titania powders published by us elsewhere [26]. The influence of various alcohols as solvents on the properties of the obtained materials and performance of utilizing them DSSCs is investigated. The activity of the prepared cells was tested under AM 1.5G simulated solar light and compared with the results of similar materials obtained employing ethanol as a solvent.

## 2. Experimental

### 2.1. Solvothermal synthesis of mesoporous TiO_2_

All chemicals were of analytical grade and were used as received without additional purification. The typical synthesis route was similar to that described by us elsewhere [26], but modified by replacing ethanol with other alcohols. In general, the syntheses were carried out as follows: 15 mL of titanium tetraisopropoxide (Aldrich) were added to 45mL of magnetically stirred alcohol (methanol (Aldrich) or n-propanol (POCh) or iso-propanol (Chempur) or n-butanol (POCh) or iso-butanol (POCh) or sec-butanol (POCh) or tert-butanol (POCh)) followed by 0.5 mL of 65% nitric acid (POCh) and 3 mL of distilled water. The solution obtained was magnetically stirred for 30 min under cover at room temperature. Colorless sol obtained was transferred to a Teflon-lined autoclave and kept in Binder FP 115 oven (Binder) in forced convection conditions at 353 K for 48 h. The pudding-like deposit was separated from the liquid via decantation and dried in forced convection conditions (Binder FP 115 oven) overnight at 353 K. The obtained solid was grounded in ball mill equipped with zirconia vial and two 13 mm zirconia balls (Mixer/Mill 8000M, Spex) and the obtained white or light-yellowish powders were labeled as TiAlk, where Alk were Me, nPr, iPr, nBu, iBu, secBu and tertBu for the synthesis carried out in methanol, n-propanol, iso-propanol, n-butanol, iso-butanol, sec-butanol or tert-butanol, respectively, as a solvent (see Table 1). The samples of TiAlk materials were subjected to heat treatment in forced convection conditions at 473 K for 2 h, with the heating ramp 1 h from room temperature to 473 K. The obtained dark brown powders were labeled as TiAlk200.

**Table 1:** The nomenclature of the synthesized materials.

Solvent	Material designation after synthesis	Material designation after calcinaton in 473 K (200°C)
Metanol	TiMe	TiMe200
n-Propanol	TinPr	TinPr200
iso-Propanol	TiiPr	TiiPr200
n-Butanol	TinBu	TinBu200
iso-Butanol	TiiBu	TiiBu200
sec-Butanol	TisecBu	TisecBu200
tert-Butanol	TitBu	TitBu200

### 2.2. Dye-sensitized devices assembling

The working electrodes for photovoltaic devices were prepared using viscous pastes spread on FTO glass (Solaronix). The viscous pastes were prepared according to the procedure described by Opara Krasovec et al. [27] and in general were made as follows: 3 mL of titanium tetraisopropoxide was added as a binding agent to ethylene glycol (Aldrich) magnetically stirred at 333 K and followed by 12.6 g of citric acid monohydrate (Aldrich). The obtained suspension was stirred and heated to 363 K and kept at this temperature until clear. The sol prepared was mixed with 5.6 g of particular TiAlk materials and ground in agate mortar for 1 h and after that the pastes were spread on FTO glass followed by annealing in Nabertherm L 5/12/P330 oven (Nabertherm) at 723 K for 2 h. As prepared electrodes, after cooling down, were immersed overnight in 10^−4^ M ethanolic (Aldrich) solution of N719 dye (Solaronix) with addition of co-adsorbent– 10^−4^ M chenodeoxycholic acid (Aldrich). FTO glass plates covered with thin platinum film were used as counter electrodes and the cells were sealed with 25 μm meltonix foil (Solaronix), which also plays a role of a spacer between the electrodes. The electrolyte (a mixture of 0.6 M 1-propyl-3-methyl-imidazolium iodide (Aldrich), 0.03 M iodine (Aldrich), 0.1 M guanidine thiocyanate (Aldrich) and 0.5 M 4-tert-butylpiridine (Aldrich) in acetonitrile (Aldrich)) was injected into the cell through two holes predrilled in the counter electrode. The final sealing was made using another piece of meltonix and microscope cover glass. The active area in a typical DSSC was approximately 0.2 cm^2^. The cells were labelled using the names of the TiAlk materials used to assemble them, so the cell utilizing TiMe material was labeled TiMe, TiiPr material gives cell called TiiPr etc. etc. Five cells representing each type were prepared and the results obtained for the best ones are presented.

### 2.3. Materials and devices characterization

Small and wide angle X-ray diffraction measurements (XRD) were performed on D8 Advance diffractometer (Bruker) with Cu Kα (λ = 0.15406 nm) radiation, the diffuse reflectance UV-Visible spectra (DRUV-Vis) were recorded on Jas.co V650 spectrometer (Jas.co) equipped with 150 mm sphere, Fourier transform infrared (FTIR) spectra were recorded on an IFS-66/s spectrometer (Bruker) using KBr powder as diluent. Nitrogen adsorption–desorption isotherms were collected using a Nova 1200e sorptometer (Quantachrome Instruments), the differential thermal analysis (DTA) and thermogravimetric analysis (TG) spectra were recorded on a Netzsch STA 409 thermogravimeter (Netzsch) heated in air up to 973 K at 4 Kmin^-1^, the morphology and particle size were examined using a Jeol JEM1400 transmission electron microscope (TEM) (Jeol). Elemental analysis was performed with a Vario EL III (Elementar) device. The photovoltaic performance of the cells obtained was measured using SS50AAA Solar Simulator (Photo Emission Technologies) equipped with an AM 1.5G filter, with the light intensity adjusted at 100 mWcm^-2^. J-V curves and electrochemical impedance spectra (EIS) were recorded on Autolab 308 Potentiostat Galvanostat (Metrohm). Incident photon-to-current efficiency measurements (IPCE) were made on Bentham PVE300 EQE/IPCE (Bentham) device.

## 3. Results and Discussion

### 3.1. Materials characterization

**Fig 1:**
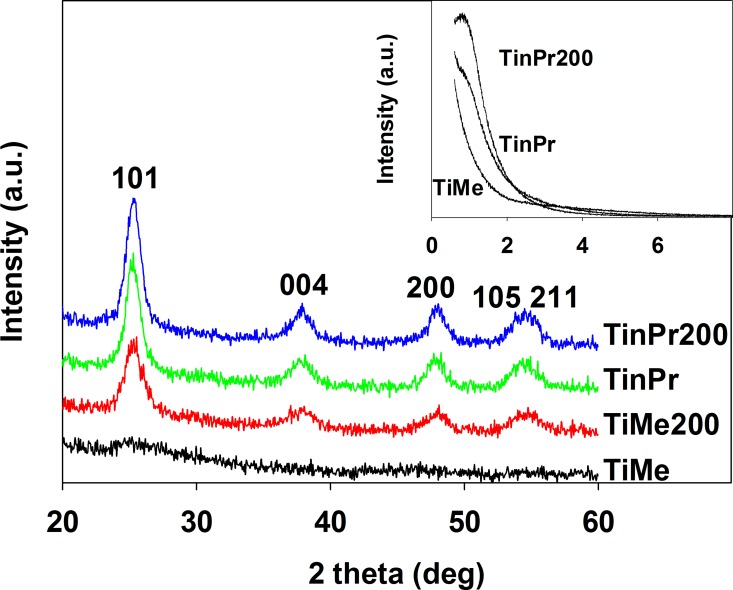
The wide angle and small angle (insert) XRD patterns of selected TiAlk samples.

The wide angle XRD measurements (Fig 1) show that a well-defined anatase structure (the peak locations are cited from the Joint Committee on Powder Diffraction Standards database; standard no. JCPDS 21–1272) is typical of all obtained samples, except for TiMe, and characteristic diffraction peaks can be observed at 2 theta 25, 38 and 48 degrees, corresponding to (1 0 1), (0 0 4) and (2 0 0) reflexes, respectively. The broad diffraction peaks observed in the 54–55 degrees range correspond to (1 0 5) and (2 1 1) reflexes, respectively. The XRD spectra of TiMe show very week signals, its crystallinity is very poor and it should be considered as amorphous titania. After temperature treatment of TiMe at 473 K, the TiMe200 sample shows a well-defined anatase structure as other samples studied. On the other hand, the annealing of the other materials caused only slight differences in their XRD patterns and, in general, only insignificant improvement of their crystallinity was observed [23, 28]. The average crystallite size calculated from the full width at half maximum of the diffraction peaks according to the Sherrer equation was in the 6.2 – 6.8 nm range for all materials; the temperature treated materials have slightly bigger crystallites than their non-treated counterparts.

The insert of Fig 1 shows the small angle XRD patterns obtained for selected TiAlk samples. A weak broad diffraction peak at around 1 degree can be observed in the diffraction patterns of TinPr200, TiiPr200, TinBu200, TitBu, TitBu200 and TisecBu200 suggesting the existence of locally ordered mesoporous titania structures in these materials [11]. The slight inflection observed in the diffraction patterns of TinPr, TiiPr, TinBu, TiiBu, TiiBu200 and TisecBu suggests that the mesopores order is not fully developed. When compared with the above mentioned materials with locally ordered mesopores, it clearly appears that in these materials the mesoporous structure is still forming during the post-synthesis temperature treatment along with anatase crystalline framework improvement in all materials. The diffraction patterns of the TiMe and TiMe200 materials showed neither diffraction peaks nor inflection(s).

**Fig 2:**
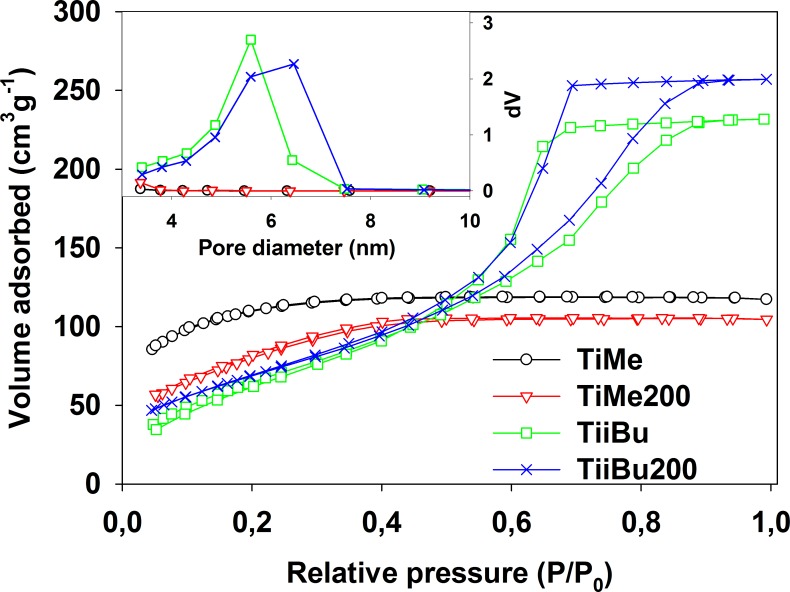
N_2_ adsorption-desorption isotherms and BJH pore distribution plots (insert) of selected TiAlk samples.

Nitrogen adsorption-desorption measurements were performed to confirm the mesoporous structure of the materials. The results are presented in Fig 2 and collected in Table 2. The specific surface areas were determined according to the BET (Brunauer–Emmet–Teller) method from N_2_ adsorption–desorption measurements; the average pore diameters and volumes were determined by applying the BJH (Barrett–Joyner–Halenda) formula on the desorption branch. Except for TiMe and TiMe200 samples, type IV isotherms with a hysteresis loop between 0.4 and 0.9 relative pressure were observed. This kind of isotherms is characteristic for mesoporous materials and this supports the conclusion that a mesoporous structure was formed in most TiAlk materials [29, 30] and is in good agreement with small angle XRD results. TiMe and TiMe200 materials were characterized by type I isotherms with relatively high surface area, 383.1 and 297.4 m^2^∙g^-2^, respectively. These properties can be explained as resulting from a highly complex microporous structure in the two above materials [29, 30]. The BJH pore distribution diagrams, presented in the insert of Fig 2, show the typical Gaussian shape for all materials, except for TiMe and TiMe200, suggesting the formation of uniformly sized mesopores in most of the obtained TiAlk materials [6]. All presented materials showed increased average pore sizes and volumes after the temperature treatment (see Table 2), possibly reflecting changes in their crystal structure. Moreover, pore diameter and volume values analysis leads to the conclusion that the materials synthesized using n-alcohols have significantly smaller pores than those prepared using branched alcohols as solvents. This effect may be connected with the more bulky shape of branched alcohol molecules which play a semi-templates role and support the formation of bigger pores [30].

**Table 2:** Textural properties of TiAlk materials.

Material	Surface area (m^2^∙g^-1^)	Pore volume (m^3^∙g^-1^)	Pore diameter (nm)	DTA peak maximum (K)	E_g_^*^ (eV)	Amount of C (wt %)
TiMe	383.1	0.181	1.840	558	3.50	6.6
TiMe200	297.4	0.162	2.174	-	3.09	1.7
TinPr	263.2	0.195	2.967	508	3.24	2.7
TinPr200	244.3	0.229	3.746	-	3.12	2.4
TiiPr	325.8	0.294	3.611	566	3.21	1.6
TiiPr200	241.4	0.317	5.256	-	3.09	1.1
TinBu	229.5	0,166	2.897	513	3.20	4.4
TinBu200	249,5	0.215	3.452	-	3.03	3.6
TitBu	303.9	0.444	5.840	517	3.19	2.4
TitBu200	271.8	0.473	6.953	-	3.15	0.9
TiiBu	252.4	0.358	5.681	512	3.22	2.8
TiiBu200	253,2	0.397	6.281	-	3.14	2.1
TisecBu	315.7	0.338	4.285	503	3.19	3.1
TisecBu200	247.0	0.348	5.642	-	3.12	1.8

* Calculated from the diffuse reflectance UV-Vis spectra using the Kubelka-Munk function

**Fig 3:**
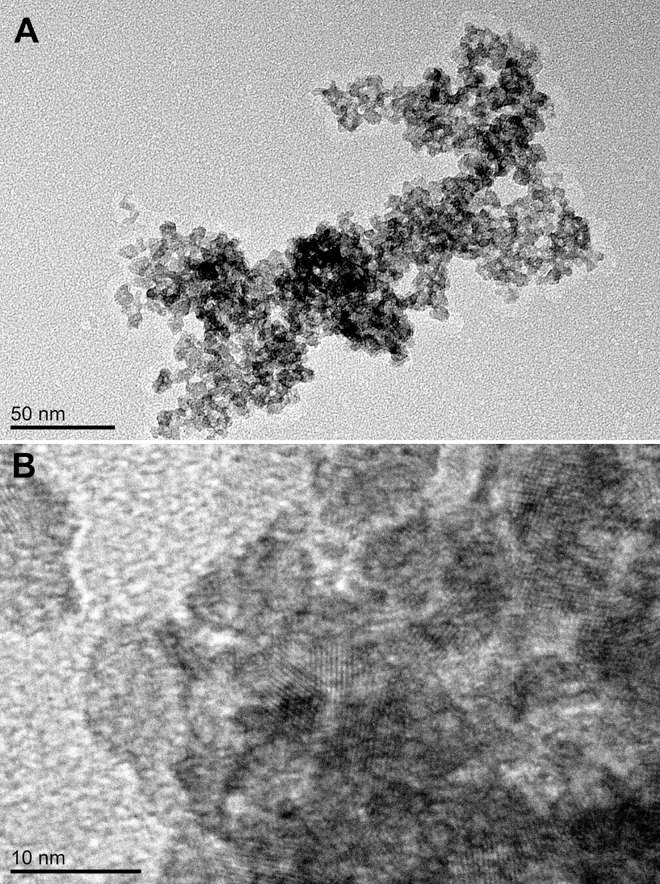
**TEM images** of the TinPr200 (A) and TitBu200 (B) materials.

Fig 3A presents the TEM image of the TinPr200 material, showing a disordered wormhole-like framework of well-defined mesopores. The mesoporous structure is built by linked and well-formed anatase crystallites, the lattice fringes of which are clearly visible; mesopores are formed between neighboring crystallites (Fig 3B). Such type of structure is typical for most of the presented materials, except for TiMe, for which only a low number of crystallites’ lattice fringes can be recognized. The observed pore and crystallites sizes are, in general, in good agreement with the above described nitrogen adsorption-desorption results and with the values calculated through the Scherrer equation, respectively, for all TiAlk materials.

**Fig 4:**
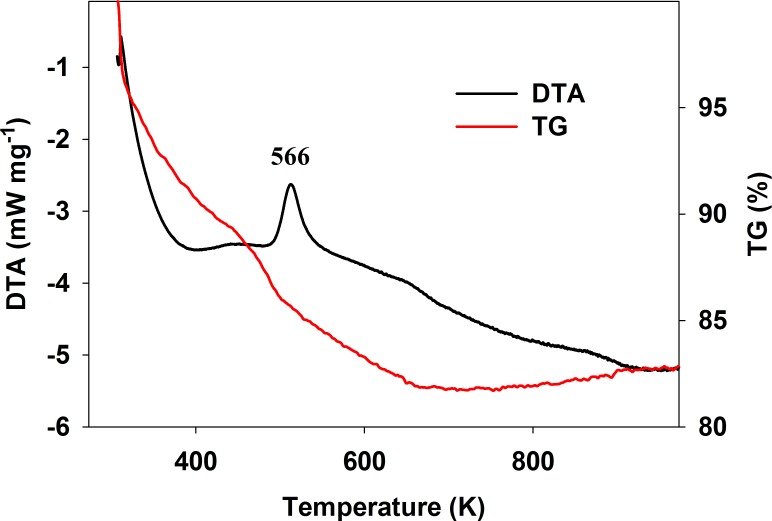
DTA and TG diagram of TiiPr material.

DTA and TG analyses were performed to investigate the influence of thermal treatments on the presented TiAlk materials; exemplary results are presented in Fig 4. The DTA and TG profiles were similar for all TiAlk materials and the most characteristic features, i.e. a strong endothermic effect accompanied by a mass loss below 400 K followed by a strong exothermic effect also accompanied by mass loss with a maximum in the 500–570 K range (depending on the material–see Table 2), were observed in all of them. The endothermic effect is the result of the evaporation of post-synthesis solvent residues physisorbed on the materials surface and/or occluded in the materials pores. On the other hand, the exothermic effect results from the thermal decomposition of the alcoxy groups existing on the surface of the as-prepared TiAlk materials.

**Fig 5:**
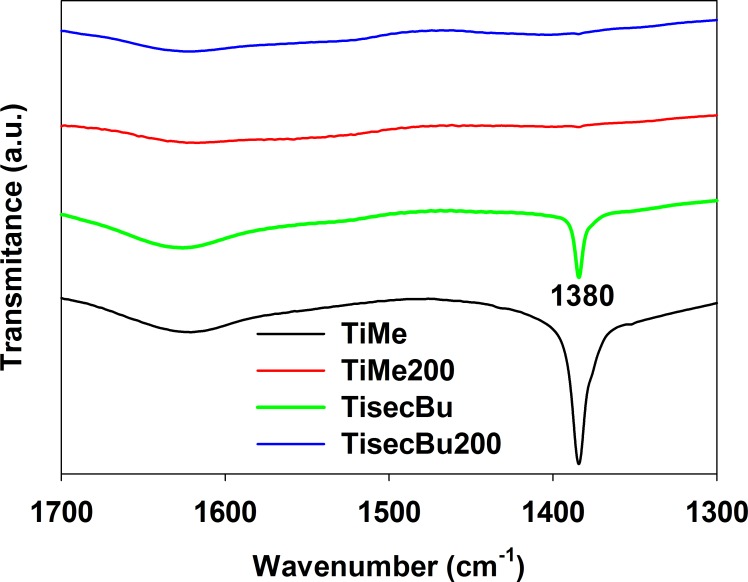
FT-IR spectra of exemplary TiAlk samples.

To confirm this interpretation, FTIR experiments were made, the results of which are presented in Fig 5. All FTIR spectra of bare TiAlk materials show the characteristic sharp intense bands at around 1380 cm^-1^, which are typical δ of C-C bonds in alcoxy groups [31]. The presence of a similar band in the spectrum of TiMe suggests that methanol is not substituting the isopropoxyl groups of titanium tetraisopropoxide used as a titanium source in the synthesis of all TiAlk materials. All these bands cannot be observed in the spectrum of TiAlk200 which supports the above conclusion on the nature of the exothermic effect observed in DTA profiles. This effect also originates brown color of TiAlk200 materials which indicates incomplete decomposition of the alcoxy groups and the formation of carbon residues on the materials surface [26]. The amount of carbon deposit was measured using elemental analysis technique and the results are collected in Table 2. The amount of carbon varied between 0.9 and 6.6% for TitBu200 and TiMe materials, respectively, and in general, the carbon impurities decreased after calcinations in good agreement with the weight loss observed in TG profiles of the TiAlk samples. It can be also observed that n-alcohols form more post-synthetic residues than their branched isomers and their thermal desorption, during both the drying process at 353 K and the heat treatment at 473 K, is less effective.

**Fig 6:**
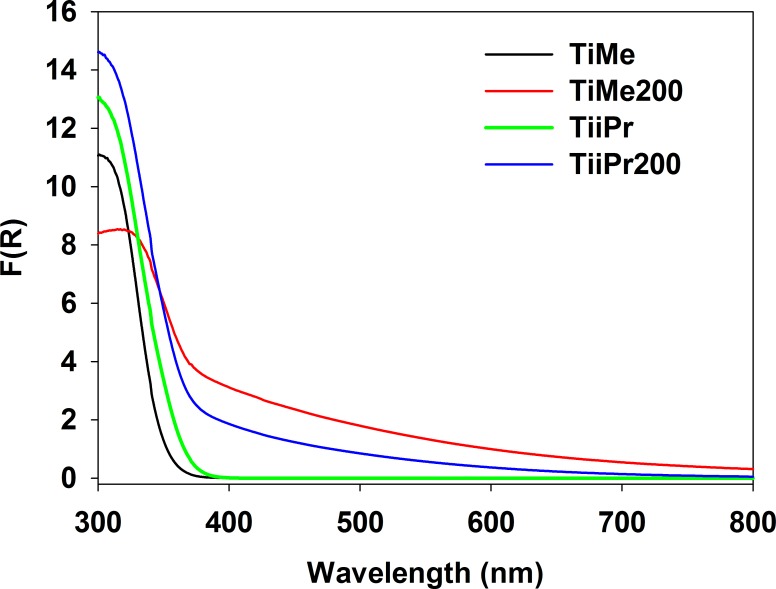
The selected diffuse-reflectance UV-Vis spectra of TiAlk materials.

The influence of carbon deposit on the properties of TiAlk materials can be observed in the diffuse reflectance UV-Vis spectra (see Fig 6). The spectra of TiAlk materials are typical of anatase titania. However, the spectra of TiAlk200 materials are characterized by a broad absorption in the whole visible light range. The bandgap values (*E*_*g*_) calculated from the UV-Vis spectra using the Kubelka-Munk function [32] are collected in Table 2. Bare TiAlk materials, except of TiMe, have *E*_*g*_ values in range from 3.19 to 3.24 eV for TinPr and TitBu or TisecBu materials, respectively, and such values are typical of anatase titania materials [1]. On the other hand, the TiMe material is characterized by a relatively high *E*_*g*_ value, i.e. 3.50 eV, and this can be explained as a result of the amorphous structure of TiMe [33]. The temperature treatment causes a decrease in the bandgap values of all investigated materials, with *E*_*g*_ values in the range from 3.03 to 3.15 eV for TinBu200 and TitBu200, respectively. The decrease in the *E*_*g*_ values is a natural consequence of the presence of carbon residues in all TiAlk200 materials [26] and of the additional formation of a well-defined anatase structure in the TiMe200 material [23, 33].

### 3.2. Photoelectrochemical studies of dye-sensitized devices

**Fig 7:**
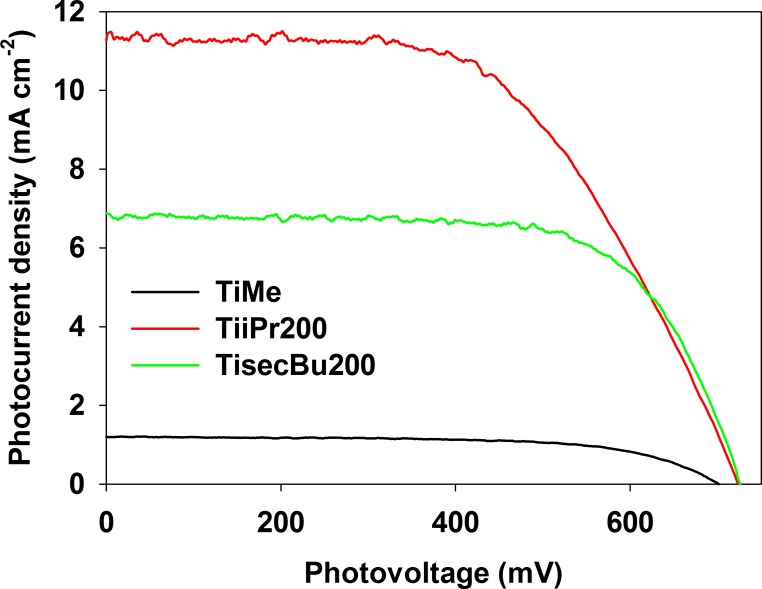
The JV curves of selected TiAlk cells.

To investigate the photoelectrochemical properties of the TiAlk materials, dye-sensitized solar cells were assembled using TiAlk materials as mesoporous anodes. The results of J-V curves measurements are collected in Table 3, together with those obtained for TiEt and TiEt200 cells [26]; exemplary curves are shown in Fig 7. The open circuit photovoltage values, V_OC_, obtained for the investigated cells are in the 704 to 763 mV range for the TiMe and TiiBu200 cells, respectively. No general tendency can be observed in V_OC_ changes between different TiAlk and TiAlk200 materials. In fact, two pairs of the TiAlk materials, TiMe/TiMe200 and TiiBu/TiBu200, are characterized by a decrease in V_OC_ value, two others, i.e. TinPr/TinPr200 and TisecBu/TisecBu200, show an increase in V_OC_ and finally the last three of them, TiiPr/TiiPr200, TinBu/TinBu200 and TitBu/TitBu200, show only slight changes in V_OC_ after the thermal treatment. The V_OC_ parameters correspond to the Fermi level of the semiconductor and the Nernst potential of the redox couple in the electrolyte [1]. So the observed changes are most probably caused by the changes in the electronic structure of particular materials.

**Table 3:** Photoelectrochemical properties of the cells assembled with electrodes prepared using TiAlk materials.

Material	J_SC_ (mA∙cm^-2^)	V_OC_ (mV)	FF (%)	η(%)N_dye_ (10^−8^ mol∙cm^-2^)	
TiMe	1.2	704	63.7	0.54	1.91
TiMe200	3.7	748	66.9	1.87	2.21
TiEt*	8.2*	670*	60.6*	3.33*	5.73*
TiEt200*	11.2*	667*	58.9*	4.39*	5.73*
TinPr	4.8	751	64.0	2.30	5.87
TinPr200	5.0	723	63.1	2.30	5.69
TiiPr	6.9	724	65.1	3.25	5.82
TiiPr200	11.5	726	55.2	4.61	5.88
TinBu	3.9	723	62.3	1.76	5.42
TinBu200	5.9	719	60.5	2.56	5.39
TitBu	11.1	704	56.9	4.45	5.65
TitBu200	7.3	716	52.1	2.71	5.48
TiiBu	7.5	702	66.5	3.51	5.61
TiiBu200	5.9	763	67.2	3.01	5.61
TisecBu	8.2	760	60.9	3.77	5.59
TisecBu200	6.9	726	67.1	3.36	5.73

* Results for TiEt and TiEt200 samples have been taken from reference [26]; please note that the N3 dye was used as a sensitizer in that work.

The latter changes were confirmed by the DRUV-Vis measurements and were strongly dependent on the formation of the carbon residues during the temperature treatment of the TiAlk materials. As the nature of organic residues on the surfaces of each TiAlk materials is different, because of the use of different alcohols during the synthesis, also the carbon residues after temperature treatment may influence the Fermi level of the materials in a different way. Consequently, no general tendency can be observed.

The V_OC_ values of all TiAlk materials are higher than those obtained for reference TiEt/TiEt200 materials, an effect to be related to the use of the N3 dye for the sensitization of the TiEt/TiEt200 materials instead of the N719 dye, the bis-tetrabutylammonium salt of N3 being more effective in light harvesting than the N3 dye [34–36].

The photocurrent density values J_SC_ obtained for TiAlk cells are in the wide range extending from 1.2 to 11.5 mA∙cm^-2^ for TiMe and TiiPr200 cells, respectively. The changes in J_SC_ values, as for V_OC_ values, do not show a general tendency. The temperature treatment of the TiMe, TinPr, TiiPr and TinBu materials results in an increase in the photocurrent density, but the temperature treatment of all materials synthesized with other butanols, i.e. TiiBu, TisecBu and TitBu, causes a significant decrease in J_SC_ values. The short circuit current is mainly influenced by two factors, i.e. the dye loading and the charge recombination/electron transport properties of the photoanode [37–40]. The relatively poor photocurrent generation of TiMe and TiMe200 cells, when compared to that of other cells presented in this work, can be easily explained by the relatively low amount of adsorbed dye on the surface of the porous electrode (see N_dye_ values collected in Table 2), while the behavior of the other TiAlk cells is more complex. The dye loading on the electrodes utilizing TinPr to TisecBu200 prepared materials reveal only slight differences and are similar to those obtained for the TiEt materials and to those which can be found in the literature [26, 41, 42]. This, in turn, indicates that the origin of the different behavior of these cells results from charge recombination/transport phenomena.

The J_SC_ results correspond to the E_g_ values changes between bare TiAlk and TiAlk200 materials. The cells in which the pairs of materials are used and in which the change in E_g_ values between as prepared and calcined ones is significant, show also an increase in J_SC_. On the other hand, the small differences in E_g_ values between the materials of each pair result in a decrease in J_SC_ after the temperature treatment. Thus the formation of carbon residues in the TiAlk materials has strong influence on their charge transport and recombination ratio properties, but the nature of these phenomena is not clear and needs more detailed studies. However, the comparison of the TiAlk cells photocurrent density values with those obtained for TiEt and TiEt200 cells shows that only two of them, i.e. TiiPr200 and TitBu, gave comparable results. Considering the use of less active N3 dye in the TiEt cells, the general conclusion is that TiAlk cell are less active than TiEt ones.

The fill factor, FF, values, representing the energy loss related to the inherent resistance of a photovoltaic device [43, 44], are in the range from 52.1% to 67.2% (see Table 2) for TitBu200 and TiiBu200, respectively. The FF values are comparable to those obtained for TiEt cells and have a minor influence on the performance of the cells.

The overall photon-to-current conversion efficiency η, which is the most important parameter of photovoltaic devices, was calculated for the presented cells, using the above described parameters. The so obtained values are collected in Table 2. The best performances of 4.61% and 4.45% were found for the TiiPr200 and TitBu cells, respectively, which is the natural consequence of the relatively high photocurrent and photovoltage values obtained for these cells. These cells are also a little bit more effective than the TiEt200 one, but this is of course a natural consequence of the use of the more active N719 dye and especially of the significantly higher V_OC_ values.

**Fig 8:**
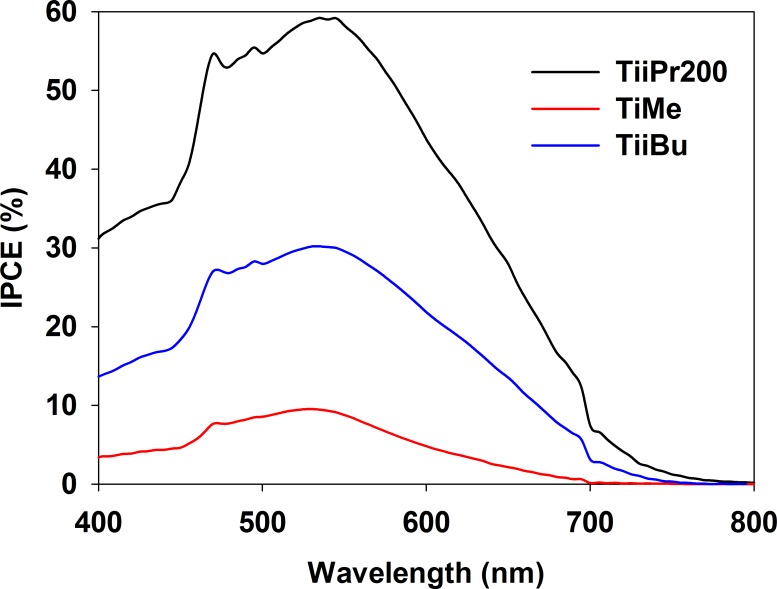
The IPCE action of selected TiAlk cells.

The IPCE profiles (exemplary curves are presented in Fig 8) have the typical shape obtained for N719 dye sensitized cells and correspond to the visible light absorption spectra of the sensitizer. The energy conversion efficiency depends on the electrode material used (see Table 4 were the IPCE values at 535 nm, which is the MLCT absorption band maximum of N719, are collected). These values are in good agreement with the η values obtained for the investigated cells.

**Table 4:** Electrochemical impedance parameters and IPCE action at 535 nm of TiAlk cells.

Material	IPCE_535_ (%)	R_CT_ (Ω)	τ_r_ (ms)
TiMe	9.5	438.5	4.3
TiMe200	26.1	287.1	5.7
TiEt	51.6	53.19	13.2
TiEt200	74.2	63.29	17.5
TinPr	25.8	109.9	9.9
TinPr200	33.8	85.0	5.7
TiiPr	43.8	50.2	13.2
TiiPr200	59.3	35.7	13.2
TinBu	34.5	148.2	7.5
TinBu200	40.9	64.3	7.5
TitBu	58.1	27.1	9.9
TitBu200	58.5	123.2	7.5
TiiBu	30.3	53.2	13.2
TiiBu200	40.3	115.5	7.5
TisecBu	61.6	56.4	13.2
TisecBu200	39.4	84.1	9.9

**Fig 9:**
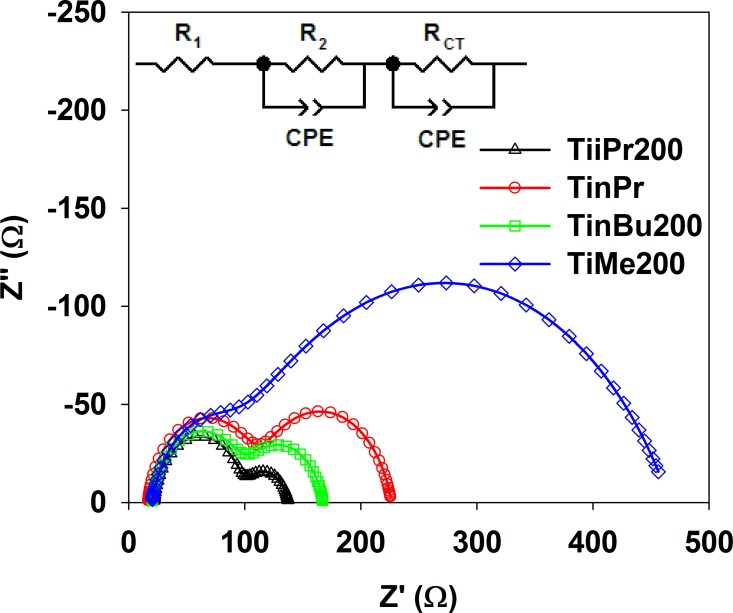
Nyquist plots of impedance spectra of DSSC devices based on different TiAlk photoelectrodes.

To better understand the reasons for such performance of the presented cells, electrochemical impedance spectroscopy (EIS) measurements were made under standard AM1.5G solar irradiation and V_OC_ forward bias conditions in the frequency range from 0.1 to 5 kHz. Typical EIS results presented as Nyquist plots (see Fig 9) consist of the following main elements: (i) the ohmic serial resistance, R_1_, in the high frequency region representing the conductive FTO electrode resistance; similar values, in the 18–22 Ω range, were found for all cells irrespective of the TiAlk material used; (ii) the redox charge transfer resistance at the FTO/Pt electrode/electrolyte interface, R_2_, observed in the high-frequency region; the values measured for TiAlk cells vary between 80 and 100 Ω, which can be explained by small differences, e.g. in Pt film thickness or discontinuities, between the counter electrodes used in the TiAlk cells [45, 46]; (iii) the TiO_2_/dye/electrolyte charge transfer resistance, R_CT_, in the low frequency region; these values were found the most variable in TiAlk cells.

The R_CT_ values obtained by fitting the impedance spectra of TiAlk cells, using an equivalent circuit showed as an insert to Fig 9, are collected in Table 4. It is clearly seen that the cells with poor efficiencies have significantly higher R_CT_ values than those with relatively high performance and this phenomenon can be explained by the differences in electron transfer impedance and charge transfer rate across the TiAlk electrodes [47]. When the cell is working under V_OC_ conditions the electrons injected into the porous semiconducting electrode should recombine at the TiO_2_/dye/electrolyte interface because the current is not passing in the external circuit [47, 48].

In such a situation the injected electron lifetime, τ_r_, can be estimated from the equation τ_r_ = (2π*f*_max_)^-1^, where *f*_max_ is a maximum frequency of the mid-frequency peak at the Bode phase plot [49, 50]. The estimated injected electron lifetime values for TiAlk cells are collected in Table 3 and are in the range from 4.3 to 17.5 ms for TiMe and TiEt200 cells, respectively. It is evident that the TiAlk cells with longer electron lifetime are more active in the photon-to-current conversion process. The injected electron lifetime has a significant influence on the recombination with the electrolyte and in general the longer electron lifetime the smaller recombination reaction rate and, consequently, the higher the photoelectric process efficiency. These two processes, charge transfer resistance and injected electron lifetime, seem to provide a good explanation of the behavior of the DSSCs assembled with use of TiAlk materials. However, as for the VOC and JSC values, no tendency can be found in the changes of the charge transfer resistance and injected electron lifetime for the various TiAlk materials.

## 4. Conclusions

The alcothermal template-free synthetic way to obtain well defined mesoporous titania presented in this work can be a useful tool for the preparation of photoactive materials. All obtained materials were successfully utilized as porous electrodes in DSSC devices and were found to be active in photovoltaic energy conversion process. The properties of the obtained materials can be easily tuned by post-synthesis treatments and change of the reaction solvent. Unfortunately, all presented cells show lower or only insignificantly higher efficiency than those prepared in the presence of ethanol. We can thus conclude that, there is no economic and ecologic reason to synthesize the template-free titania materials for DSSC applications using alcohols other than ethanol, which is cheap and can be produced using environmental friendly technologies.
